# Positivity Effect and Working Memory Performance Remains Intact in Older Adults After Sleep Deprivation

**DOI:** 10.3389/fpsyg.2019.00605

**Published:** 2019-03-22

**Authors:** Andreas Gerhardsson, Håkan Fischer, Mats Lekander, Göran Kecklund, John Axelsson, Torbjörn Åkerstedt, Johanna Schwarz

**Affiliations:** ^1^Department of Psychology, Stockholm University, Stockholm, Sweden; ^2^Stress Research Institute, Stockholm University, Stockholm, Sweden; ^3^Department of Clinical Neuroscience, Karolinska Institutet, Stockholm, Sweden

**Keywords:** sleep deprivation, positivity bias, emotion, older adults, sustained wakefulness, working memory, executive functions, affect

## Abstract

**Background:** Older adults perform better in tasks which include positive stimuli, referred to as the positivity effect. However, recent research suggests that the positivity effect could be attenuated when additional challenges such as stress or cognitive demands are introduced. Moreover, it is well established that older adults are relatively resilient to many of the adverse effects of sleep deprivation. Our aim was to investigate if the positivity effect in older adults is affected by one night of total sleep deprivation using an emotional working memory task.

**Methods:** A healthy sample of 48 older adults (60-72 years) was either sleep deprived for one night (*n* = 24) or had a normal night’s sleep (*n* = 24). They performed an emotional working memory *n*-back (*n* = 1 and 3) task containing positive, negative and neutral pictures.

**Results:** Performance in terms of accuracy and reaction times was best for positive stimuli and worst for negative stimuli. This positivity effect was not altered by sleep deprivation. Results also showed that, despite significantly increased sleepiness, there was no effect of sleep deprivation on working memory performance. A working memory load × valence interaction on the reaction times revealed that the beneficial effect of positive stimuli was only present in the 1-back condition.

**Conclusion:** While the positivity effect and general working memory abilities in older adults are intact after one night of sleep deprivation, increased cognitive demand attenuates the positivity effect on working memory speed.

## Introduction

While older age is usually characterized by impairments in cognitive functions such as working memory (Salthouse and Babcock, 1991; Clarys et al., 2009), older adults show a sustained or even improved ability when it comes to emotion regulation compared to their younger counterparts (Nashiro et al., 2012). Ample evidence confirms this modulation of emotional processing in older adults that is characterized by a shift in attention from negative to positive stimuli, termed *the positivity effect* (for a recent review, see Carstensen and Deliema, 2018). However, studies show that this positivity effect can be modulated by stress induced by aversive movie clips (Everaerd et al., 2017) and cognitive demand (Reed et al., 2014). Thus, this suggests that the age-related preference for positive stimuli (or decreased attention toward negative stimuli) may be attenuated when healthy older adults are exposed to additional challenges. One such challenge may be sleep loss. With a steadily growing older adult population, there may be requirements of a longer active work life, also in sectors with non-regular working hours, including challenging night work. Although older adults usually handle the effects of sleep deprivation, like impaired attention or lowered mood, better than young adults (Scullin and Bliwise, 2015; Schwarz et al., 2018a), it is not known whether the positivity effect is affected by sleep deprivation. Therefore, we here tested whether the positivity effect in older age is impacted by sleep deprivation.

The most influential theory explaining the positivity effect is the socioemotional selectivity theory (SST), which suggests that older adults prioritize positive and avoid negative experience due to the notion of a decreasing time horizon (Reed and Carstensen, 2012). The positivity effect has not only been demonstrated for behavioral choices but has been linked to neural changes such as a shift in amygdala activity from increased activity to both positive and negative stimuli in young adults to increased activity to positive stimuli alone in older adults when rating their arousal from observing emotional pictures (Mather et al., 2004). Some studies also suggest that there is an age-difference in brain network recruitment for memory encoding of positive and negative images (Addis et al., 2010; Ziaei et al., 2017). This shift in attention for emotional stimuli has also further implications for cognitive functions like memory. In memory tasks, older adults usually perform better on positive stimuli compared with negative or neutral stimuli, whereas the opposite is found for younger adults (Carstensen and Mikels, 2005; Reed et al., 2014). As for working memory some studies show an age-related behavioral positivity effect (Mammarella et al., 2013; Truong and Yang, 2014) whereas others do not (see Murphy and Isaacowitz, 2008). However, failing to find a positivity effect has been attributed to task constraints such as instructions to use specific encoding strategies which restrict the older participants from using innate strategies (Reed et al., 2014; Ziaei and Fischer, 2016). Even when the positivity effect was not evident in direct measures of accuracy and reaction times, using diffusion modeling, Spaniol et al. (2008) could tie the positivity effect to mnemonic processes rather than a response bias toward positive stimuli. Moreover, a recent meta-analysis synthesized the age-related positivity effect on working memory and concluded that there is a relatively stable though small effect on accuracy, but a somewhat stronger effect for reaction times (Schweizer et al., 2018). Taken together, although the positivity effect is well established there is so far limited research on every day activities that could affect the positivity effect in older adults.

According to the SST the positivity effect is goal-directed and motivationally guided and not a bi-product of cognitive decline – therefore the positivity effect can be modulated by cognitive demand (Reed and Carstensen, 2012). One study showed that the positivity effect was not only attenuated but reversed when a distracting task was introduced together with viewing emotional pictures (Mather and Knight, 2005). Similarly, another study showed that older individuals with higher executive attention scores had a stronger positivity effect for memories of public events (Petrican et al., 2008). Besides cognitive demands, stress may attenuate the positivity effect. It was recently shown that inducing acute stress by watching aversive movie clips increased the neural activity in visual processing areas and amygdala in older adults, making the neural response more similar to one of younger adults’, thus suggesting an attenuation of the positivity effect (Everaerd et al., 2017). Taken together, these studies suggest that older adults’ tendency to guide attention toward positive and away from negative stimuli may be sensitive to challenging conditions such as increased cognitive demand and acute stress.

In the young adult population sleep deprivation has been associated with lapses in attention, reduced working memory performance, and mood changes (Pilcher and Huffcutt, 1996; Lim and Dinges, 2010). Moreover, in young adults, lack of sleep causes alterations in emotional processing (Tempesta et al., 2018), primarily by increased reactivity to emotional stimuli and an impaired ability to regulate emotional reactivity (Yoo et al., 2007; Gujar et al., 2011). We recently reported that sleep deprived younger adults (18–30 years), but not the controls (with full sleep), responded faster to positive and slower to negative relative to neutral pictures on an emotional working memory task, which could be interpreted as a positivity effect (Gerhardsson et al., 2019). We also found that sleep deprivation overall impaired accuracy and omission rate, but not reaction times. Interestingly, many of the detrimental artifacts of sleep deprivation seem to be less severe for older adults, but studies are largely limited to attention and less complex tasks, with non-emotional stimuli (Scullin and Bliwise, 2015).

Stress has been found to attenuate the positivity effect (Everaerd et al., 2017), at least on a neural level. This suggests that the positivity effect could be vulnerable to challenges. Sleep deprivation can induce a mild stress response, both in terms of subjective ratings and in terms of increased cortisol (Meerlo et al., 2008; Schwarz et al., 2018b). However, no prior study has investigated if the positivity effect in older adults is affected by total sleep deprivation. Our aim of the present study was twofold. As the positivity effect is modulated by challenging conditions such as cognitive demand and stress, we wanted to investigate if the positivity effect in older adults would be affected by one night of sleep deprivation. We also wanted to test if older adults’ known resilience to effects of sleep deprivation would extend to working memory in a task with positive, neutral and negative picture scenes using two levels of working memory load.

## Materials and Methods

### Participants

As part of a larger protocol (Schwarz et al., 2018b), data from 48 (32 women) healthy older adults (*M*_Age_ = 66.2 ± 3.4 years, range 60–72) that had been randomized to a sleep control (SC, *n* = 24, *M*_Age_ = 66.2 ± 3.7 years) or a total sleep deprivation (TSD, *n* = 24, *M*_Age_ = 66.1 ± 3.2 years) condition was analyzed. The full study also included young participants and a stress condition. Here we used data from the non-stressed older adults only (see Table 1 for sample characteristics). Initial screening for disease, psychiatric disorders, and sleep habits were done via an online questionnaire and participants who fulfilled the criteria were invited for an interview and some additional screening assessment, including the Mini mental State examination (Folstein et al., 1975), approximately 1 week before the experiment. The study protocol was approved by the Regional Ethical Review Board in Stockholm, and all participants signed an informed consent form.

**Table 1:** Descriptive characteristics [mean (standard deviations)] and *t*-statistics of group comparisons.

	Control	TSD	*t*-statistics	*p*
Total *n* (Male)	24 (8)	24 (8)		
*n* test time (13:00/16:00)	13/11	13/11		
Age (years)	66.21 (3.73)	66.13 (3.21)	0.08	0.934
BMI (kg/m2)	23.88 (2.75)	23.26 (2.13)	0.87	0.389
MMSE	29.17 (0.82)	28.71 (1.68)	1.20	0.238
ISI	4.00 (2.81)	3.75 (2.07)	0.35	0.728
ESS	5.96 (3.42)	6.29 (3.06)	0.36	0.723
**Actigraphy (*n* missing)**				
Sleep period 1 night before (2)	07:53 (00:54)			
Sleep start 1 night before (2)	23:18 (00:56)			
Sleep end 1 night before (2)	07:11 (00:41)			
Sleep period 2-3 nights before (5)	07:42 (01:01)	08:01 (00:43)	-1.18	0.246
Sleep start 2-3 nights before (5)	23:47 (01:02)	23:17 (00:39)	1.91	0.065
Sleep end 2-3 nights before (5)	07:29 (00:36)	07:18 (00:56)	0.75	0.457


*BMI, body mass index; MMSE, Mini Mental State Examination (Folstein et al., 1975); ISI, Insomnia Severity Index (Bastien, 2001); ESS, Epworth Sleepiness Scale (Johns, 1991); TSD, Total Sleep Deprivation.*

### Design and Procedure

The task and study protocol were the same as reported recently for younger adults (Gerhardsson et al., 2019). All participants performed a practice run of the emotional working memory task in relation to the screening interview 1 week before the experiment. They were then also instructed to keep a regular sleeping schedule and to refrain from alcohol, caffeine and exercise 24 h prior to the experiment. They were instructed to keep a sleep diary and to wear an actiwatch (Cambridge Neurotechnology^®^, Cambridge, United Kingdom), a wrist worn activity monitor that can be used to analyze sleep-wake patterns, approximately 3 days before the experiment.

Both groups started the test session in the afternoon at 13:00 or 16:00 (balanced between groups) the day after the manipulation night, with the emotional working memory task performed 2 h in to the test session. Participants in the TSD group spent their time in the laboratory from 22:00 the night before the test session until the end of the test session the following day. Most of the time during the night was spent doing leisure activities, but they also performed cognitive tests and rated their sleepiness using the Karolinska Sleepiness Scale (KSS: Åkerstedt and Gillberg, 1990). Participants randomized to the Control condition slept in their home, monitored by an actiwatch and arrived to the laboratory in time for the start of the test session. From 2 h before the emotional working memory task, the protocol was the same as for the sleep deprived participants.

### Emotional *N*-Back Task

To measure emotional working memory we used an *N*-back task with positive, negative, and neutral pictures from International Affective Picture System (IAPS: Lang et al., 2008). After excluding close-up faces and food items (see Benedict et al., 2012) the sample pictures were categorized into positive, neutral, or negative based on normative valence ratings (Lang et al., 2008; see Supplementary Table S1 for picture details) and then separated into a positive-neutral and negative-neutral block. Valences were separated to reduce the risk of spill-over effects. Starting order of the valence blocks was counterbalanced between participants. Each valence block contained a 1-back and a 3-back condition and the starting condition was also counterbalanced between participants, but kept identical across valence blocks. Which means that if for instance the positive-neutral block was first, starting with 3-back, the 1-back followed and in the subsequent negative-neutral block the 3-back was also before the 1-back. All of the 96 pictures were presented to each participant. To balance the role of the picture and have sufficient number of trials, each picture was presented three times, once as target, once as probe and once as non-target in one of the blocks, summing up to a total of 288 trials per participant, distributed over the different valence blocks, and cognitive load conditions.

A trial consisted of a stimulus, presented for 900 ms followed by an inter-stimulus interval of 1000 ms. Responses were recorded within the full trial duration (1900 ms) and participants were instructed to respond as fast and correct as possible to if the current stimulus was the same as the one *N* steps back in the sequence, by pressing the L-key for YES and the A-key for NO on the keyboard. Total task duration was approximately 12 min. The task was programmed using PsychoPy (Peirce, 2007), and performed on a laptop with a 17.3 inch display (resolution 1920 × 1080).

### Additional Outcomes

Before the emotional working memory task participant rated their sleepiness using the KSS (Åkerstedt and Gillberg, 1990) and their current affective state using the Positive Affect Negative Affect Schedule (PANAS: Watson et al., 1988).

### Statistical Analysis

Performance accuracy (d^′^), omissions and reaction times were treated as outcome variables. For the d^′^ and omissions we performed 2 × 2 × 3 mixed analyses of variance (ANOVA). Sleep condition (TSD, Control) was a between subject variable, and load (1-back, 3-back) and valence (positive, neutral negative) were within subject variables. Two participants from the TSD condition, with all missing answers in at least one cell, were removed, leaving 46 participants for the analysis. Calculations of d^′^ were done excluding omissions and adjusted for extreme proportions by adding 0.5 to the sum of each cell and 1.0 to the sum of each row (Hautus, 1995). We analyzed the log-transformed reaction times from the correct targets (hits) using mixed-effects modeling with sleep, load, and valence as fixed factors. The best fitting model included random intercepts for participant and item, and random slopes for valence and load within participant (model fitting overview see Supplementary Table S2). We trimmed the data of observations ±2.5 SD from the residual mean (1.82% removed) and refitted the model using restricted maximum likelihood estimation. Satterthwaite degrees of freedom approximation were used to estimate ANOVA-style contrasts from the final model (Table 2). As we were interested in evaluating if the performance of the TSD condition was comparable with the performance of the control group we complemented the traditional null-hypothesis testing with a Bayesian approach, evaluating the main contrasts of interest with Bayesian *t*-tests that estimates a Bayes factor (Morey and Rouder, 2018). The Bayes factor is a likelihood ratio in favor of one hypothesis over the other given the data (Dienes and Mclatchie, 2018). In traditional statistical inference one usually calculates the probability of the data given a null hypothesis of no difference between the conditions and decides to reject or keep the null hypothesis based upon the *p*-value. However, if we keep the null hypothesis we cannot distinguish if there is evidence for no difference or if there is no evidence to speak of (Dienes and Mclatchie, 2018). A Bayes factor of 1 indicates no evidence in either direction. If testing the alternative hypothesis against the null (BF_10_), as we have done here, a value above 1 supports the alternative hypothesis and a value below 1 is in support for the null-hypothesis. The strength of evidence is commonly labeled moderate if the BF_10_ is above 3 or below 0.33, and strong if BF_10_ is above 10 or below 0.1 (Beard et al., 2016). Thus, in order to directly address whether; (a) older adults’ were resilient to sleep deprivation, and (b) whether any positivity effect remained intact after sleep deprivation, we complemented the initial analysis with Bayesian *t*-tests on each outcome with a non-informative Jeffreys prior (*r* = 0.707) (Morey and Rouder, 2018). Analysis was performed using R (R Core Team, 2016). Results are reported as mean ± standard deviations if not stated otherwise.

**Table 2:** ANOVA style contrast table of outcome variables with *F*-statistics.

	d^′^		Omissions (%)		RT (log)	
	*F (df) P*	*p*	*F (df)*	*p*	*F (df)*	*p*
sleep	0.44 (1, 44)	0.513	0.84 (1, 44)	0.364	2.67 (1, 43)	0.11
load	**162.13 (1, 44)**	**<0.001**	3.07 (1, 44)	0.087	**237.56 (1, 45.1)**	**<0.001**
valence	**5.48 (2, 88)**	**0.006**	0.08 (2, 88)	0.927	**3.71 (2, 61.8)**	**0.03**
sleep × load	0.04 (1, 44)	0.843	0.43 (1, 44)	0.518	2.31 (1, 45.1)	0.135
sleep × valence	0.1 (2, 88)	0.902	0.1 (2, 88)	0.908	1.71 (2, 62.9)	0.189
load × valence	1 (2, 88)	0.371	0.38 (2, 88)	0.682	**5.25 (2, 3251)**	**0.005**
sleep × load × valence	1.65 (2, 88)	0.198	2.51 (2, 88)	0.087	0.14 (2, 3237.2)	0.865


*RT, reaction Time. The degrees of freedom (*df*) for RT was estimated using Satterthwaite’s method, using a mixed-effects model design without aggregated means. Significant effects are marked in bold font.*

### Supplementary Analysis

In order to evaluate the differences between the age groups, in terms of susceptibility to sleep loss and positivity effect we performed a *post-hoc* analysis using the present data and data from the previously published article (Gerhardsson et al., 2019). As this was not part of any of our main questions, specifications for that analysis and results can be found in the Supplementary Material.

## Results

**FIGURE 1:**
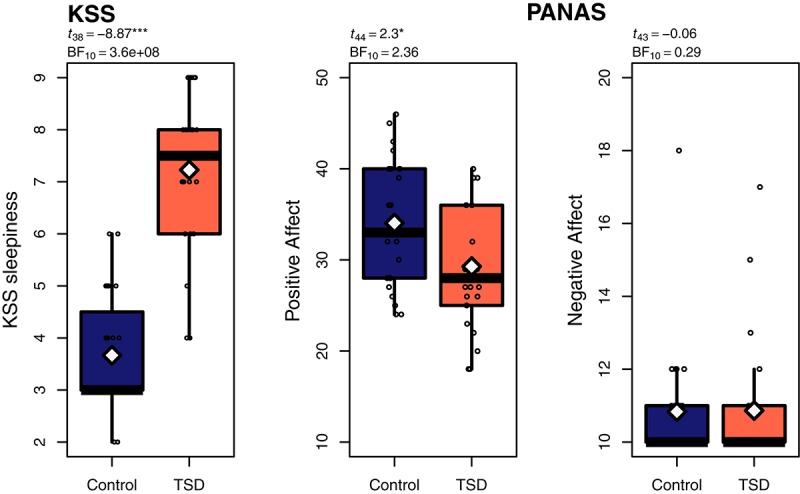
Boxplots of Karolinska Sleepiness Scale (KSS: Åkerstedt and Gillberg, 1990) and Positive Affect Negative Affect Schedule (PANAS: Watson et al., 1988); white diamonds representing the means. *p*-values based on Welch *t*-tests. ^∗^*p* < 0.05, ^∗∗^*p* < 0.01 ^∗∗∗^*p* < 0.001.

A Welch *t*-test showed that the sleep deprived group was significantly sleepier (7.2 ± 1.5) than the sleep control group (3.7 ± 1.1) as measured before the task by KSS (*t*_38_ = 8.87, *p* < 0.001, BF_10_ > 3.6^e+08^), see Figure 1. Welch *t*-tests for each of the valences of the PANAS ratings showed that the sleep deprived rated significantly lower on positive affect (29.3 ± 7.1) than the control group (34.0 ± 7.0, *t*_44_ = 2.30, *p* = 0.026, BF_10_ = 2.36) but there was no difference in negative affect (TSD: 10.9 ± 7.7, Control: 10.8 ± 8.0, *t*_43_ = 0.06, *p* = 0.95, BF_10_ = 0.29), see Figure 1.

**FIGURE 2:**
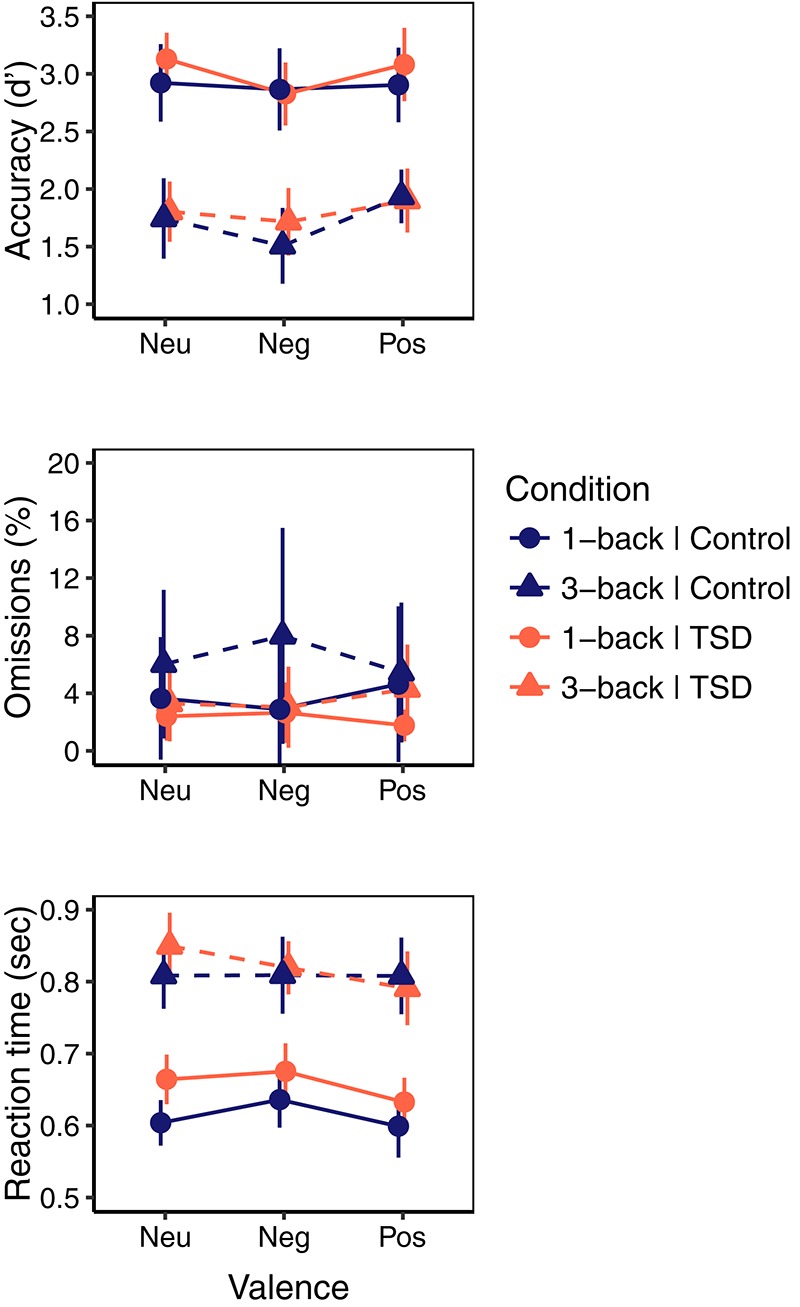
Performance on emotional *N*-back task for Control (blue) and sleep deprivation (TSD; red) condition on neutral (Neu), negative (Neg), and positive (Pos) pictures, by 1-back (circle, solid) and 3-back (triangle, dashed) load. Error bars represent 95% Confidence Interval.

**FIGURE 3:**
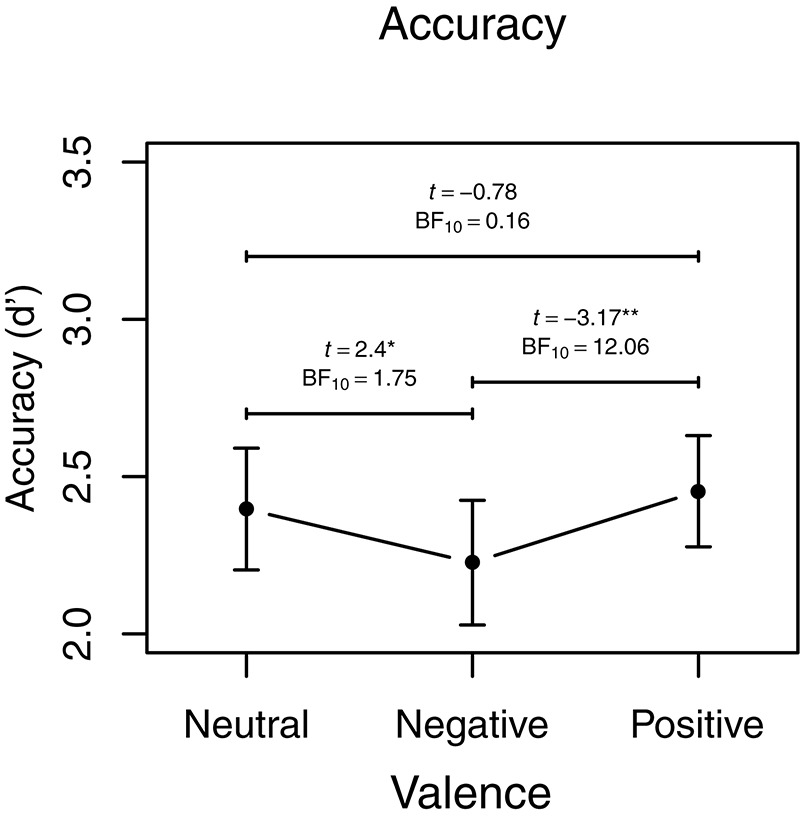
Accuracy, main effect of valence visualized with aggregated means. Error bars represent 95% Confidence Interval. BF_10_ indicate evidence in favor of the alternative hypothesis over the null. ^∗^*p* < 0.05, ^∗∗^*p* < 0.01.

For the emotional *N*-back task there were no significant main effects or interactions involving sleep deprivation (see Table 2 for ANOVA style statistics). Aggregated means of emotional working memory performance are visualized in Figure 2. For accuracy (d^′^) there was a main effect of load showing higher accuracy on the easier 1-back (2.95 ± 0.72) than on 3-back (1.77 ± 0.68). There was also a main effect of valence, with *post-hoc* comparisons indicating higher accuracy for positive (2.45 ± 0.85) compared with negative pictures (2.32 ± 0.96, *t*_88_ = 3.17, *p* = 0.002, BF_10_ = 12.06) and neutral (2.40 ± 0.93) compared with negative pictures (*t*_88_ = 2.40, *p* = 0.019, BF_10_ = 1.75) but no difference between positive and neutral pictures (*t*_88_ = 0.78, *p* = 0.44, BF_10_ = 0.16), see Figure 3. There were no significant effects on omissions.

**FIGURE 4:**
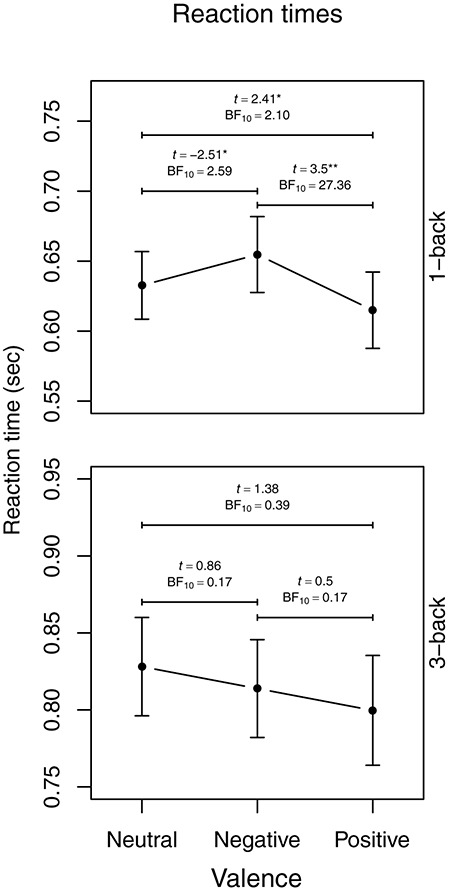
Aggregated reaction time means visualizing the load × picture valence interaction. Error bars represent 95% Confidence Interval. BF_10_ indicate evidence in favor of the alternative hypothesis over the null. ^∗^*p* < 0.05, ^∗∗^*p* < 0.01.

For reaction time, there was a main effect of load indicating faster responses on 1-back (0.631 s ± 0.178) than on 3-back (0.812 s ± 0.208). A main effect of valence indicated faster responses to positive stimuli (0.693 s ± 0.196) than to both negative (0.718 s ± 0.194, *t*_51_ = 2.44, *p* = 0.018, BF_10_ = 2.24) and neutral stimuli (0.709 s ± 0.201, *t*_61_ = 2.53, *p* = 0.013, BF_10_ = 2.61) but no significant difference between neutral and negative pictures (*t*_59_ = -0.89, *p* = 0.38, BF_10_ = 0.21), indeed the Bayes factor indicates support for no difference in reaction times between neutral and negative pictures. A significant load × valence interaction indicated that this effect of valence was present in the 1-back condition with faster responses to positive (0.611 s ± 0.092) than to neutral (630.74 s ± 0.081, *t*_50_ = 2.41, *p* = 0.020, BF_10_ = 2.10) and negative pictures (0.651 s ± 0.096, *t*_44_ = 3.50, *p* = 0.001, BF_10_ = 27.36) and faster responses to neutral than to negative pictures (*t*_51_ = 2.51. *p* = 0.015, BF_10_ = 2.59), while there was no difference in the 3-back condition (*F*_2,88.68_ = 1.14, *p* = 0.33), see Figure 4.

The Bayes factor for the comparison between sleep deprivation and the control condition indicated weak evidence in favor of the null hypothesis, on d^′^ (BF_10_ = 0.35), omissions (BF_10_ = 0.41) and reaction times (BF_10_ = 0.57), suggesting that there was little difference in performance between the sleep conditions, see Supplementary Figure S1. In the same manner we tested the difference between sleep deprived and controls by each valence. All Bayes Factors, except for neutral reaction times (BF_10_ = 2.00), were in favor for the null hypothesis with a range of 0.32–0.42, i.e., that sleep deprivation did not affect the response to any valence, see Supplementary Figure S2. To get an idea about the strength of evidence for a positivity effect in the control condition and the sleep deprivation condition separately we also tested the positive against the negative pictures on the means aggregated over working memory load separate for each sleep condition for d^′^ and reaction times. Those comparisons showed that the positivity effect was weak for reaction times (BF_10_ = 2.39) and accuracy (BF_10_ = 2.10) in the sleep deprived group, and no evidence to speak of in the control group on reaction times (BF_10_ = 0.86) and accuracy (BF_10_ = 1.34).

## Discussion

Here we investigated the effect of sleep deprivation on emotional working memory in older adults and our results showed that responses to positive pictures were more accurate and faster than to negative pictures, regardless of sleep condition. Thus, the positivity effect seems resilient to sleep deprivation. Moreover, the results showed that one night of sleep deprivation did not adversely affect the overall working memory performance. Our findings are in line with two established theories with regard to older adults, namely that they perform better toward positive stimuli and worse toward negative stimuli (Carstensen and Deliema, 2018), and that they are resilient to effects of sleep deprivation (Scullin and Bliwise, 2015). On the other hand, the positivity effect was not present for reaction times in the high cognitive load condition, suggesting that cognitive demand may play a moderating role.

A main aim of the present study was to investigate if the positivity effect in older adults would be affected by one night of sleep deprivation. The results confirm the expected positivity effect with faster reaction times and more accurate responses for positive compared with negative pictures. This effect remained intact after sleep deprivation. Overall, the findings of a positivity effect in older adults corroborates a bulk of evidence showing an age-related positivity effect in attention (Mather and Carstensen, 2003), memory (Carstensen and Mikels, 2005; Reed et al., 2014), and working memory (Mammarella et al., 2013; Truong and Yang, 2014; Schweizer et al., 2018). Given that sleep deprivation leads to a mild upregulation of autonomic and neuroendocrine stress systems (Meerlo et al., 2008; Schwarz et al., 2018b) our results are at odds with those of Everaerd et al. (2017). They found induced stress to be associated with increased amygdala activation in older adults when presented with emotional facial expressions, making the activation more similar to that of young adults suggesting a reduced positivity effect on a neural level. Compared to Everaerd et al. (2017) there are some differences in study design and outcomes to consider. While sleep deprivation increases the basal activity of the stress system (Schwarz et al., 2018b), the increase is rather small in comparison to the more acute stressor of aversive movie clips. Another aspect to consider is the use of task, where we employed a working memory task with emotional pictures scenes, Everaerd et al. (2017) used passive viewing of fearful and happy facial expressions with no apparent intention of a comparison between the expressions, but data showed no difference between fearful and happy in the older age group in neither condition. Also instead of positivity effect measured through neural activity (Everaerd et al., 2017), we used performance on a working memory task as an indicator of positivity effect and found no differences between sleep deprivation and rested control condition. While we should be careful to not speculate too much about underlying neural mechanisms without having such measures, it is possible that sleep deprivation increased the neural response to negative images (Yoo et al., 2007), and positive images (Gujar et al., 2011), while not affecting the performance on the working memory task. Lastly and importantly, besides being a mild stressor and alter emotional functioning, sleep deprivation impacts attention and cognitive functions such as working memory (Lim and Dinges, 2010), and the results should be interpreted within this context. Research on the emotion-cognition interaction after sleep deprivation, and especially on older adult samples, is scarce. The results from the present study are behavioral and neural imaging could help to pinpoint the underlying mechanisms which could explain why older adults maintain a positivity effect after sleep deprivation.

Another observation is that subjective positive affect (measured using the PANAS) was reduced, while the positivity effect, in terms of working memory performance, was not affected by sleep deprivation. This could indicate that the mood component was separate from emotional processing of external stimuli, in line with the notion that the positivity effect is a goal-related process, relying on cognitive resources (Reed et al., 2014). Others have found that inducing positive mood increases working memory performance in older adult (Carpenter et al., 2013), and while sleep deprivation reduced positive mood in the present study, it might not have been enough to cause a decline in working memory performance. Moreover, the impact of sleep deprivation on the reward network activation (Gujar et al., 2011) together with our recent findings of improved working memory speed on positive pictures (Gerhardsson et al., 2019) for young adults, could have prompted us to expect an increased rather than a decreased positivity effect in older adults. One explanation to that no such change was observed after sleep deprivation could be that the positivity effect reached a limit with the older adults already at a rested state.

We complemented our analysis with Bayesian *t*-tests for the differences between the valences. With this procedure we were able to directly estimate the evidence for an effect against the evidence for no effect, instead of only rejecting the null hypothesis as we would have done with traditional null-hypothesis testing. We found that there was strong evidence for the positivity effect (negative-positive) on the whole group on accuracy (BF_10_ = 12.06), weak evidence for a positivity effect on reaction times (BF_10_ = 2.24), and strong evidence for reaction times in the 1-back condition (BF_10_ = 27.36). This means that the presence of an effect given the data is 12.06 and 2.24 (27.36 for 1-back) times more likely than the absence of an effect. Additionally, we separated the groups and tested the positivity effect on sleep deprived and controls, respectively. Here the evidence was weak for a positivity effect on reaction times and accuracy in the sleep deprived group, while in the control group there were indications of no evidence (BF_10_ close to 1), which from a Bayes factor perspective indicates that there is too little or too noisy data (Dienes and Mclatchie, 2018). The analysis likely suffered from the reduction of sample size to half as was done when the groups were analyzed separately. Given the absence of a sleep × valence interaction and that the evidence is weak after separating the groups, we should be careful in our interpretation, but they may give a hint of that the positivity effect could even increase for older adults after sleep deprivation.

Similar to previous research on general cognitive performance in older age (Scullin and Bliwise, 2015) we found that emotional working memory performance in older adults remained largely intact after sleep deprivation. The sustained ability to perform after sleep loss was evident for both accuracy and reaction time, indicating that there was no trade-off between speed and accuracy. While much of previous research on the age effect of sleep deprivation is restricted to attention tasks our findings add to previous research by further showing that the increased working memory demand and emotional content do not impair the working memory ability after sleep deprivation in older adults. As for the positivity effect we tested the main effect of sleep condition for all outcomes using Bayesian *t*-tests. For all outcomes related to the sleep deprived against control contrasts we found evidence, although weak, in favor of the *no difference* (null) hypothesis as indicated by the BF_10_ being below 1 and close to 1/3. The same was true for the comparison between the conditions by valence, again with weak support of the null hypothesis, with one exception; the reaction times to the neutral pictures were slower in the sleep deprived group. With no correction for multiple comparisons and tests based on aggregated data, this effect should be interpreted with caution, but it could indicate that emotional content, irrespective of valence can aid in working memory performance after sleep loss, perhaps through increased neural reactivity (Yoo et al., 2007; Gujar et al., 2011). Compensatory neural activation, reduced accumulation of sleep pressure or low need for sleep are some of the theories proposed to explain older adults’ sustained performance after sleep deprivation (Scullin and Bliwise, 2015). In the present study, both groups had good habitual sleep indicating that a state of chronic sleep deprivation likely cannot explain our results. The sleep deprived participants reported being significantly sleepier than the rested participants and the sleep deprived group also had significantly higher cortisol levels as we have reported recently (Schwarz et al., 2018b), confirming that the resilience to sleep deprivation in older adults does not cover all aspects.

Although sleep deprivation did not alter the positivity effect, increased cognitive demand attenuated the effect on working memory speed. That is, whereas there was strong evidence in favor of a difference in reaction time between negative and positive images in the 1-back condition that requires less cognitive resources, there was no difference between the valences in the 3-back condition that requires more cognitive resources. Although we did not find the positivity effect to be fully reversed (Mather and Knight, 2005), additional cognitive demand increased the reaction times to positive pictures to a level similar to negative and neutral pictures. The collective evidence suggests that task constrains, such as increased cognitive demand, attenuate the positivity effect (Reed et al., 2014). This indicates that the positivity effect is to some extent a goal-directed process that relies on cognitive resources as proposed by the SST (Reed and Carstensen, 2012). To the best of our knowledge this is the first study to investigate the positivity effect on visual working memory using emotional pictures with two levels of cognitive load in older adults. However, more research is needed to dissociate the executive components involved in the age-related positivity effect.

A number of limitations need to be considered. Besides actiwatches, we did not have any objective sleep measures to evaluate sleep quality in detail. However, actiwatch, sleep diary and other sleep measures showed no difference between the groups before the sleep manipulation night. Moreover, many older adults suffer from chronic sleep problems and other health issues and those were not included in the present study, thus we should be careful before generalizing our results to the general older population. There is large variability in sleep, health and cognitive functioning among older adults and more research is needed that cover different aspects of the effect of sleep loss and gives more attention to a growing elderly population. Although we found resilience in older adults to effects of sleep deprivation on working memory it is possible that a longer duration of sleep deprivation or extended task duration could increase the demand and have an impact on the emotional working memory performance. Another aspect worth considering is that, although the emotional stimuli used was sufficient to elicit an effect of valence, the ecological relevance of the procedure could be questioned. More ecologically valid emotional material, such as film clips or real situations is warranted to fully understand the positivity effect in older adults after sleep deprivation. One strength of the study is that we adapted a mixed-effects model on the reaction times that considered the individual variability within each load and valence and the picture, improving generalizability of the sample of pictures to the population of picture stimuli.

## Conclusion

The present results show that sleep deprivation does not alter the positivity effect on working memory, while higher cognitive demand caused an attenuation of the positivity effect for reaction times but not accuracy. This suggests, that the positivity effect in older adults remains for some, but not all, demanding conditions. Overall, working memory accuracy and reaction times are not affected by a full night of sleep deprivation in older adults. These findings can contribute to a broadening of the view on aging including some positive aspects, and by improving the understanding regarding both the positivity effect under pressure and regarding the general consequences of sleep deprivation on emotional working memory in an older population.

## Data Availability

The datasets generated for this study are available on request to the corresponding author.

## Author Contributions

AG, HF, ML, GK, JA, TÅ, and JS contributed to the study design, interpretation of results, and preparation of manuscript. AG and JS additionally contributed to the data collection and AG performed the data analysis.

## Conflict of Interest Statement

The authors declare that the research was conducted in the absence of any commercial or financial relationships that could be construed as a potential conflict of interest.
