# Supplementary material for: Positivity Effect and Working Memory Performance Remains Intact in Older Adults After Sleep Deprivation
Source: Front Psychol. 2019 Mar 22;10:605. doi: 10.3389/fpsyg.2019.00605 (PMC6440387; doi:10.3389/fpsyg.2019.00605)
Supplement: Supplementary file 1 [file Data_Sheet_1.pdf]

Supplementary Material

Positivity Effect and Working Memory Performance Remains Intact in Older Adults After Sleep Deprivation

Andreas Gerhardsson*[1,2], Håkan Fischer[1], Mats Lekander[2,3], Göran Kecklund[2,3], John Axelsson[2,3], Torbjörn Åkerstedt[2,3] & Johanna Schwarz[2,3]

[1]Department of Psychology, Stockholm University, Stockholm, Sweden
[2]Stress Research Institute, Stockholm University, Stockholm, Sweden
[3]Department of Clinical Neuroscience, Karolinska Institute, Stockholm, Sweden

*** Correspondence:** Andreas Gerhardsson: andreas.gerhardsson@psychology.su.se

Table S1. IAPS normative valence and arousal scores (Lang et al., 2008).

	Negative	Positive	Neutral
N	24	24	48
Valence			
Mean (SD)	2.62 (0.26)	7.74 (0.30)	5.17 (0.46)
Range	2.06–2.99	7.25–8.28	4.13–5.97
Arousal			
Mean (SD)	4.93 (0.57)	4.63 (0.64)	3.84 (0.82)
Range	4.00–6.61	3.73–6.28	2.51–5.51

Note: IAPS: International Affective Picture System. Picture reference number. Negative: 2301, 2750, 2751, 2799, 6300, 9000, 9180, 9181, 9220, 9280, 9291, 9295, 9320, 9342, 9415, 9430, 9435, 9560, 9561, 9830, 9832, 9922, 9925, 9927. Positive: 1440, 1441, 1460, 1463, 1721, 1750, 1811, 1920, 1999, 2091, 2154, 2314, 2345, 2530, 5202, 5210, 5480, 5780, 5829, 5830, 5831, 7502, 7580, 8190. Neutral: 1121, 1350, 1505, 1560, 1616, 1908, 2191, 2597, 5130, 5395, 5535, 5731, 5740, 5900, 7002, 7012, 7016, 7025, 7032, 7035, 7041, 7045, 7061, 7081, 7096, 7100, 7130, 7137, 7161, 7186, 7188, 7205, 7211, 7224, 7236, 7248, 7496, 7497, 7504, 7512, 7513, 7547, 7560, 7600, 7820, 7830, 8325, 9913

Table S2. Overview of model fitting for linear mixed effects models for reaction time

Model including fixed factors: sleep × load × valence *(tested against)*	*df*	AIC	BIC	LL	χ^2	df_{χ^2}	*p*
1. Random intercept: subject	14	-841.96	-755.87	434.98			
2. Random: subject and picture (1)	15	-840.93	-748.69	435.47	0.975	1	.323
3. Random: subject and picture, slope for load within subject (2)	17	-964.58	-860.04	499.29	127.65	2	< .001
4. Random: subject and picture, slope for valence within subject (2)	20	-847.07	-724.08	443.53	16.14	5	.006
**5a. Random: subject and picture, slope for load within subject and slope for valence within subject (3)**	**23**	**-971.37**	**-829.94**	**508.69**	**18.79**	**6**	**.005**
**5b. Random: subject and picture, slope for load within subject and slope for valence within subject (4)**	**23**	**-971.37**	**-829.94**	**508.69**	**130.30**	**3**	**< .001**

Note: AIC: Akaike's Information Criterion, BIC: Bayesian Information Criterion, LL: Log-Likelihood. Model 5a/b (bold) best fit. Covariance(s) between the random intercept and random slope was included. P-values are for the likelihood ratio test.

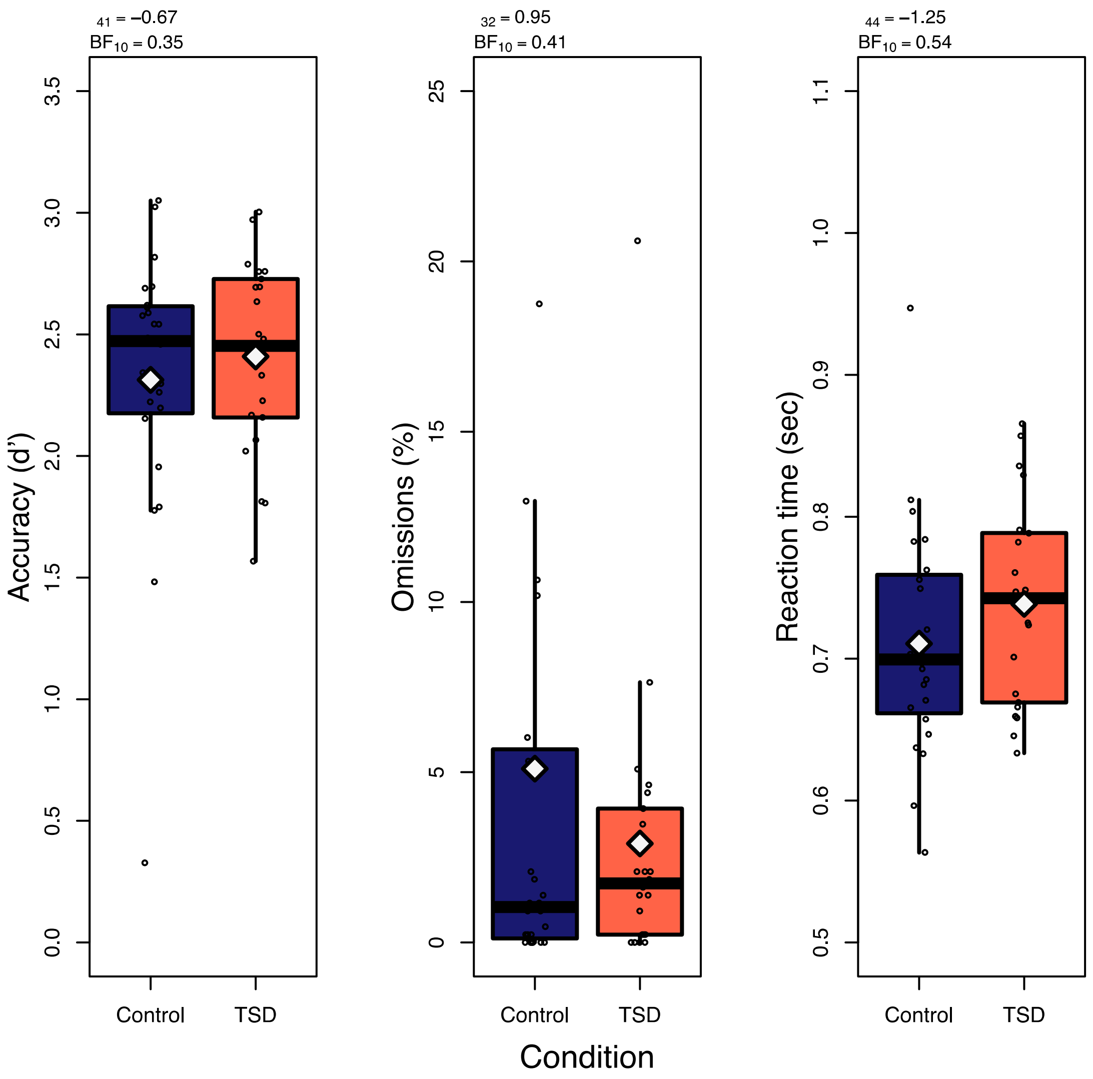

Figure S1. Boxplots of sleep deprived (red) and controls (blue), collapsed over N-back load and valence. Diamonds show means. Top row shows *t*-statistics and Bayes Factor for the alternative (BF_{10}) hypotheses, testing the difference between control and sleep deprived (TSD) participants on aggregated means.

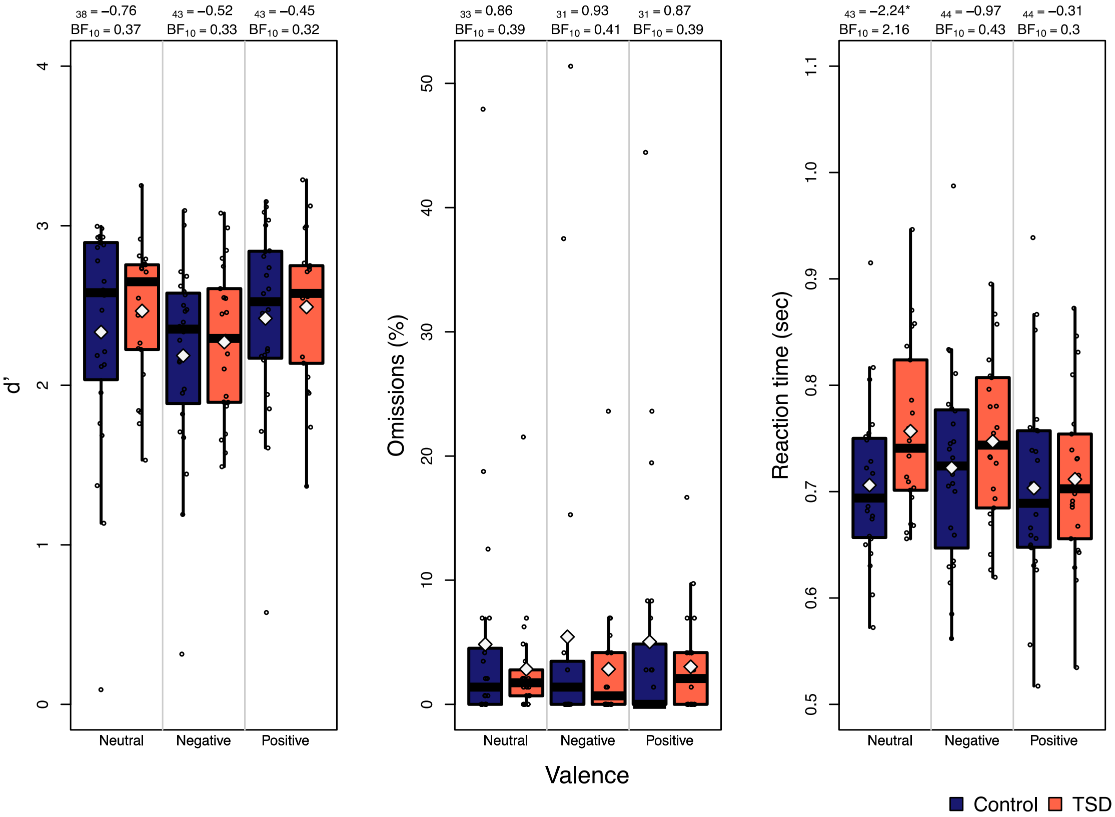

Figure S2. Boxplots of sleep deprived (red) and controls (blue) by valence, collapsed over N-back load. Diamonds show means. Top row shows *t*-statistics and Bayes Factor for the alternative (BF_{10}) hypotheses, testing the difference between control and sleep deprived (TSD) participants on aggregated means. * $p < 0.05$ (uncorrected)

Positivity effect and effect of sleep deprivation in young and older adults

To evaluate the difference between age groups, we included the data from our previously published study (Gerhardsson et al., 2019) and analyzed the d', reaction times, subjective sleepiness (KSS; Åkerstedt and Gillberg, 1990) and affective state (PANAS; Watson et al., 1988). Analysis was performed using R (R Core Team, 2016).

Results

To investigate the age group difference in susceptibility to sleep loss we collapsed the data across load and valence for each participant and performed a linear regression model with sleep and age as independent variables. We estimated the contrast to compare the young and older adults in each sleep condition, and the interaction. For d', there was no significant difference between the young and the older participants in either condition (p >.05), and Bayes factor indicate that the evidence was uncertain in both the control group (BF_{10} = 1.90), and in the sleep deprived group (BF_{10} = 0.56). A significant interaction indicated that the difference between the age groups differed between the conditions, however, the Bayes factor shows only anecdotal support (BF_{10} = 2.84). For reaction times the young adults outperformed the older adults equally in both conditions, see Figure S3A.

In order to investigate whether an age-dependent positivity effect was present in the control condition we used the control (normal night sleep) condition only and collapsed the data across load for each participant. We used a mixed linear regression model, with participant as a random factor, and estimated the contrasts between the age groups for each valence. For d', the young adults' performance was significantly better compared to the older adults on neutral, and negative pictures but not on the positive pictures. Bayes factors indicate that the evidence in favor of a difference between the groups were moderate for negative pictures (BF_{10} = 5.12), and anecdotally supporting no difference on positive pictures (BF_{10} = 0.38), whereas the evidence for the neutral pictures was more uncertain (BF_{10} = 1.04). For reaction times, the young outperformed the older adults on all valences, see Figure S3B.

In addition to the performance measures, we compared the differences in sleepiness and affective state ratings between the age groups in each condition. Extracting the contrasts from a linear regression model we found that older adults were less sleepy than the young adults in the control condition, while there was no significant age difference in sleepiness in the sleep deprivation condition. Older adults rated themselves as more positive compared to young adults in both conditions. No significant interactions or differences in negative affect was noted. See Figure S5 for full statistics.

Discussion

Taken together, these results do not fully corroborate previous findings (Scullin and Bliwise, 2015). The age × sleep interaction indicates that the difference between the groups differed between the

conditions. Thus, the difference between sleep deprivation and control conditions was larger for the young than for the older adults. However, when separating the sleep conditions the difference in performance in the control group indicated better performance in younger adults but was not significant (p = .09) and Bayes factor indicates that there is too much uncertainty to draw any conclusions in either direction. There was no significant difference in the sleep deprivation group and Bayes factor show anecdotal support for no difference. One reason for the uncertainty may be that we collapsed the load conditions, increasing the variance. For reaction times, the older adults were overall slower, which was to be expected based on previous literature (Salthouse, 1996). This was the case also when comparing the groups across different valences. For accuracy we observed a positivity effect in a sense that the performance in the negative condition was worse for the older adults compared to the young, but similar for positive pictures. These findings are in line with previous literature on the age-related positivity effect (Carstensen and Deliema, 2018). For sleepiness, the lack of an interaction effect may indicate that the older adults are less sleepy in general. A similar pattern was found for positive affect. As a concluding remark, this research question was not planned with an age compression in mind, why the results should be interpreted with caution and future studies should investigate further the age differences after sleep deprivation related to emotional and cognitive functions.

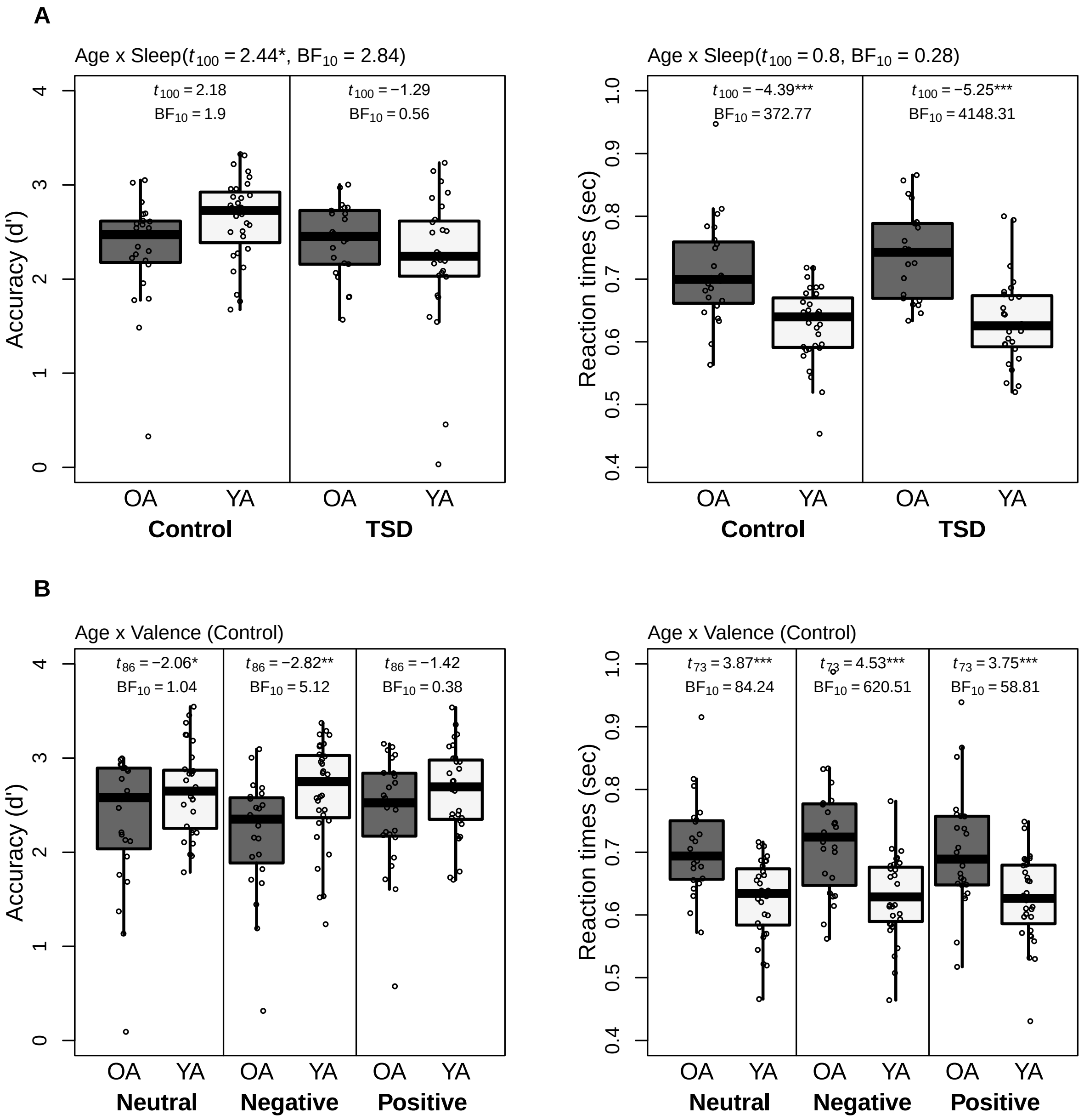

Figure S3. Reaction times and Accuracy (d') for young (YA) and older adults (OA) in Control or sleep deprivation (TSD) condition. Each plot show *t*-statistics and Bayesian *t*-test from differences between age groups in each sleep condition (A) and for control group only for each valence (B). * $p < .05$, ** $p < .01$, *** $p < .001$ (Bonferroni corrected).

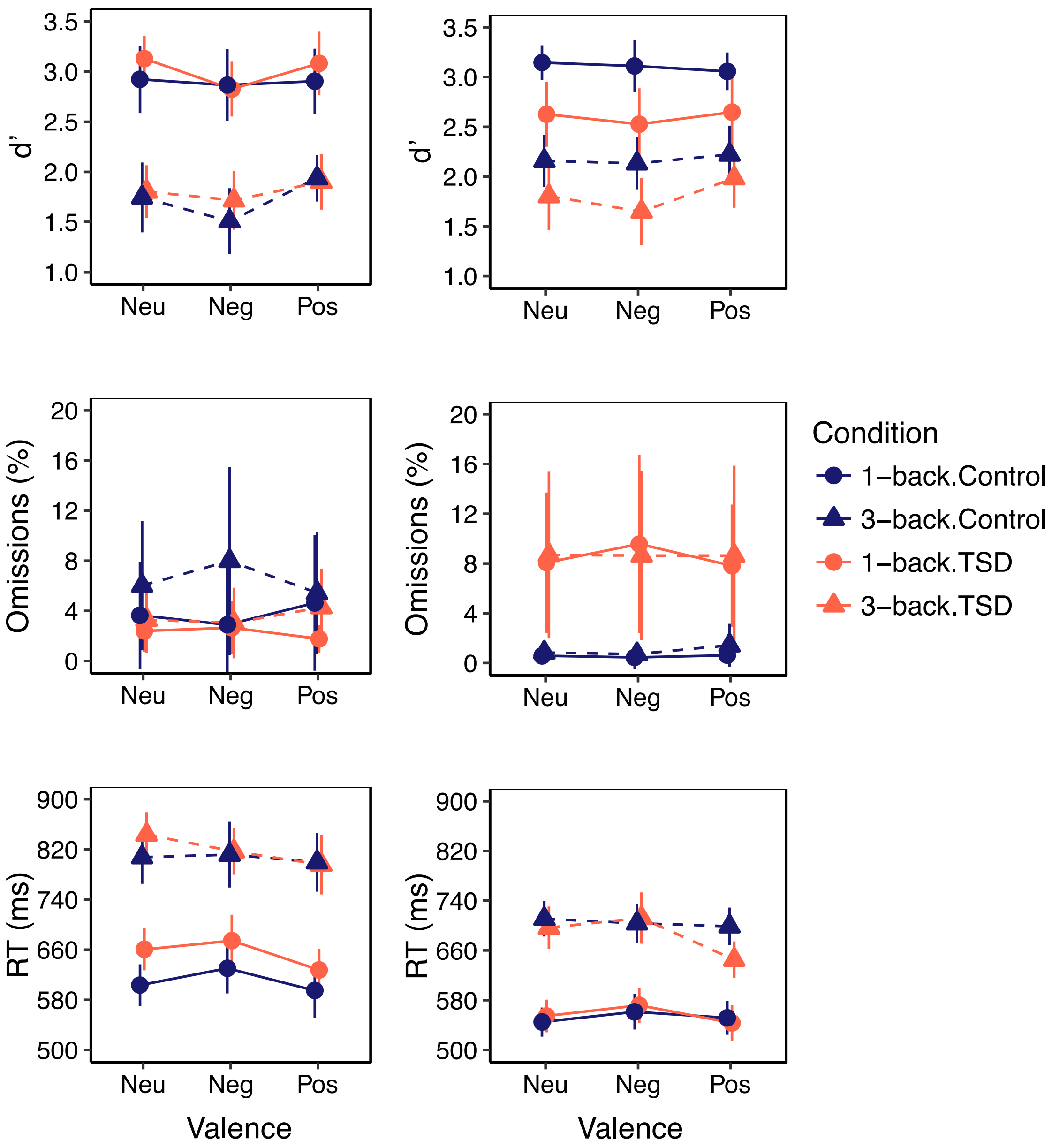

Figure S4. RT: Reaction times. Performance on emotional N-back task for Control (blue) and sleep deprivation (TSD; red) condition on neutral (Neu), negative (Neg) and positive (Pos) pictures, by 1-back (circle, solid) and 3-back (triangle, dashed) load. Young adults (right) adapted from Gerhardsson et al. (2018). Error bars represent 95% CI.

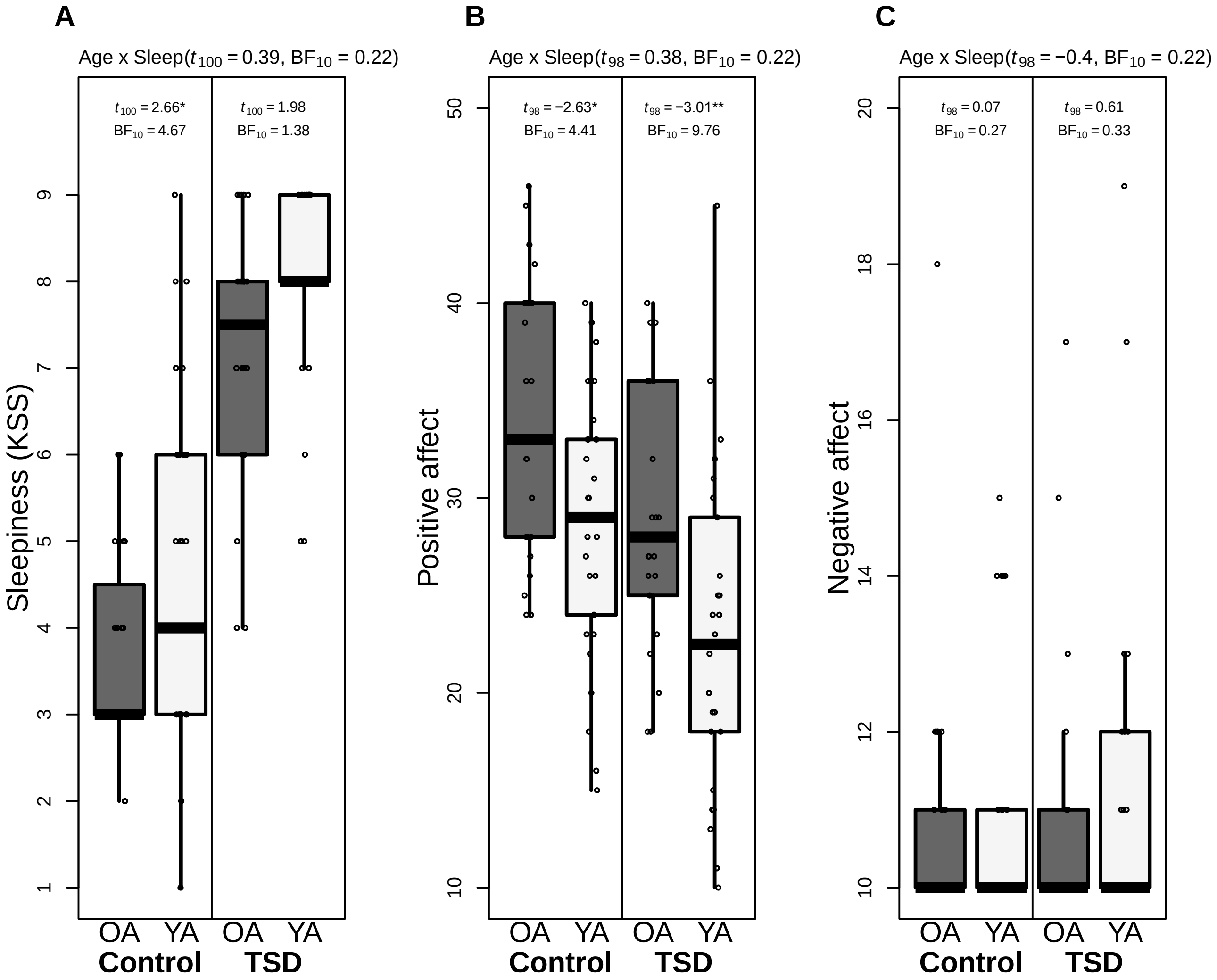

Figure S5. Boxplots of (A) sleepiness (KSS: Åkerstedt and Gillberg, 1990), (B) positive affect and (C) negative affect (PANAS: Watson et al., 1988), for young (YA) and older (OA) adults. In each plot *t*-statistics and Bayesian *t*-tests for the contrasts are presented . Above each panel, the statistics of the interaction effect. * $p < .05$, ** $p < .01$ is presented, (Bonferroni corrected).

References

Åkerstedt, T., and Gillberg, M. (1990). Subjective and objective sleepiness in the active individual. *The International journal of neuroscience* 1, 29–37. doi:10.1017/CBO9781107415324.004.

Carstensen, L. L., and Deliema, M. (2018). The positivity effect: a negativity bias in youth fades with age. *Current Opinion in Behavioral Sciences* 19, 7–12. doi:10.1016/j.cobeha.2017.07.009.

Gerhardsson, A., Åkerstedt, T., Axelsson, J., Fischer, H., Lekander, M., and Schwarz, J. (2019). Effect of sleep deprivation on emotional working memory. *Journal of Sleep Research* 28, e12744. doi:10.1111/jsr.12744.

Lang, P. J., Bradley, M. M., and Cuthbert, B. N. (2008). International affective picture system (IAPS): Affective ratings of pictures and instruction manual. Technical Report A-8. Gainesville, FL.: University of Florida.

R Core Team (2016). R: A language and environment for statistical computing.

Salthouse, T. A. (1996). The processing speed theory of adult age differences in cognition. *Psychological Review* 103, 403–428. doi:10.1037/0033-295X.103.3.403.

Scullin, M. K., and Bliwise, D. L. (2015). Sleep, Cognition, and Normal Aging. *Perspectives on Psychological Science* 10, 97–137. doi:10.1177/1745691614556680.

Watson, D., Clark, L. A., and Tellegen, A. (1988). Development and validation of brief measures of positive and negative affect: The PANAS scales. *Journal of Personality and Social Psychology* 54, 1063–1070. doi:10.1037/0022-3514.54.6.1063.
